# Magnetohydrodynamic unsteady natural convection slip flow in a vertical parallel plate microchannel heated with constant heat flux

**DOI:** 10.1016/j.heliyon.2024.e41502

**Published:** 2024-12-26

**Authors:** Mohsen Saghafian, Mehdi Moslehi, Omid Ali Akbari

**Affiliations:** a Department of Mechanical Engineering, Isfahan University of Technology, Isfahan, 84156-83111, Iran; b Department of Mechanical Engineering, Faculty of Engineering, Arak University, Arak 38156-88349, Iran

**Keywords:** MHD, Natural convection, Unsteady, Slip flow, Microchannel, Magnetic field, Electrically conducting fluid

## Abstract

This research presents a numerical study over the unsteady natural convection of an electrically conducting fluid in an open-ended vertical parallel plate microchannel under uniform and asymmetric heat flux subjected to a uniform lateral magnetic field. Slip velocity, as well as temperature jump at channel walls, are modeled using a first-order model. The effects of Knudsen number)*Kn*(, heat flux ratio)rq(, Grashof number)*Gr*(, and Hartmann number)M(on mass flow rate, the maximum temperature of the wall, and average Nusselt (*Nu*) as a function of time are discussed. In this research, the flow in the limit of 0≥ M ≥ 2; 0≥*Kn* ≥ 0.1; 0.1≥ rq ≥ 1, and 0.0001≥ L ≥ 0.01 were simulated numerically. Furthermore, and study is conducted on steady-state velocity and temperature profiles. The research showed that increasing the *Ha* value, causes a reduction in the mass flow and an increase in the maximum temperature of the wall with respect to time. As M increases, the average Nusselt number as a function of time diminishes. Also, these variables present at least one overshoot or undershoot in time. The overshoot and undershoot decrease with increasing *Ha*. It is also revealed that the magnetic force effect decreases the fluid flow and thermal behavior with an increase in *Kn*. In addition, in higher *Gr*, magnetic force influence on profiles of velocity and temperature decreases for all rq and *Kn* values. Moreover, the magnetic field has no significant effect on the time at which steady state condition is attained for all rq and *Gr* number values.

## 1 Introduction

Microfluidic system devices and micro-electro-mechanical systems (MEMS) have been interesting so much in these years due to developments in microfabrication technologies and frequent applications in all branches of science and engineering. Some of these applications are drug delivery, chemical reactors, and biotechnology [[1], [2], [3], [4], [5]]. The MHD force and natural convection slip flow are utilized for microelectrochemicall cell transport, micro heat exchangers, nuclear reactors, oil exploration, geothermal energy extraction, and plasma studies [[6], [7], [8], [9], [10], [11]]. In a large portion of these usages, it is interesting to control the motion of fluid, mix or separate fluid, and control the temperature profile [[12], [13], [14]]. Magnetohydrodynamic (MHD) [[15], [16], [17], [18]] provides a suitable and flexible way for this purpose [[19], [20], [21], [22], [23], [24], [25], [26]]. The main parameter to realize the fluid flow regime is known as *Kn*. This parameter could be defined as the ratio of fluid mean free path to the characteristic length scale fluid flow domain. As reported in Refs. [27,28], until the *Kn* is lower than (Kn≤ 0.001), the continuum approach is acceptable while for (0.001< Kn <0.1), Navier-stocks equations are useable with modified boundary Conditions. This region's flow is called the slip flow regime. For large values of Knudsen (*Kn* ≥ 10), the flow is assumed to be free molecular. In this study, the slip flow regime is considered for analyzing thermal characterizations as well as the fluid flow. Lots of study works have been done corresponding to forced convection gaseous slip flow in microgeometry as shown in Refs. [29,30]. However, few parts of these studies are carried out on the mixed convection and free convection gas flows in microscale. Hamza et al. [31] proposed an exact solution mathematical model for evaluating the influence of magnetohydrodynamics on the unsteady fluid flow in an exothermic reaction flown in a vertical channel with H-spacing. They obtained speed, temperature, heat transfer rate, and skin friction coefficient through solving the mathematical model. Their research result showed that as the Darcy number (Da) increases, the speed will rise as well. Another finding of this research showed that the velocity profile diminishes for increasing the magnetic field parameter (*Ha*). Jamalabadi et al. [32] studied flow characteristics as well as heat transfer of an incompressible and conductive fluid flow in a microchannel in the existence of a transverse magnetic field. They investigated the governing Navier-Stokes and energy equations of magnetohydrodynamic flow to achieve a better understanding of the impact of wall presence (friction and heat transfer) and flow properties (temperature and velocity). They concluded from their findings that as the *Ha* increases, the sinusoidal profile of velocity becomes smoother and the magnetic field applied to the flow plays the role of a resistance force against the fluid flow in natural convection. Furthermore, few studies have been conducted on MHD forced and mixed convection. Previous studies related to MHD mixed convection flow investigated the flow over a horizontal plate.

Muhammad et al. [33] conducted numerical work to study the natural convection magneto-hydrodynamic flow in a vertical concentric cylinder subjected to an induced magnetic field. In their study, they derived shell friction, mass flux, and induced current density formulations. Their results indicated that heat absorption, induction current density, induction magnetic field, and speed parameters show a negative trend with increasing *Ha* value. Moreover, skin friction and mass flux numerical values in the cylindrical walls increase (decrease) as the heat absorption parameter increases. Generally speaking, this value's behavior decreases as *Ha* increases. Reddappa et al. [34] made an effort to study the well-developed laminar, steady mixed convection slip flow in a vertical parallel plate microchannel while the heat flux of channel walls is asymmetric.

They showed that as *Ha* increases, the velocity near the cold wall rises as well; however, the condition is oppositely different for the hot wall. Ozturk [35] studied the steady laminar MHD slip flow inside a rectangular microchannel under uniform heat flux analytically. They found that as the Hartman number increases, the Nusselt number increases as well. Aina and Malgwi [36] devoted themselves to an analytical study concentrating on the effect of the lateral magnetic field as well as the effect of suction/injection on the natural MHD convection flow of a conductive fluid in a sloping porous microchannel. Considering the conditions of velocity slip and temperature jump in porous channel walls, they investigated the velocity and temperature characteristics. They concluded that the effect of the inclination angle on the velocity of the fluid depends on the wall ambient temperature difference ratio. Therefore, increasing the inclination angle leads to an increase in the velocity of the fluid in the porous microchannel for some picked wall ambient temperature values. Microchannel thermal exchangers are used in a few vital and different areas: aerospace; car; bioengineering; cooling of gas turbine edges, control and prepare businesses; refrigeration and discuss conditioning; infrared finders and effective laser mirrors and superconductors; microelectronics; and thermal control of film testimony. For example, microchannel cold plates are progressively becoming fundamental within the plan and administration of electric vehicle (EV) battery warm administration frameworks. Mechanical engineers and battery pack respectability depend on these innovative liquid cooling arrangements to preserve ideal temperatures, make strides in execution, and expand the life expectancy of EV batteries. Understanding the complexities of microchannel cold plate plan is vital for successful EV battery warm administration framework integration.

According to the best researcher's knowledge, it seems that the MHD unsteady free convection gaseous slip flow in a vertical plate microchannel heated by uniform and asymmetric heat flux is still to be explored in the literature. This research, the pioneering research on this topic, aims to study the influences of magnetic field and gas rarefaction on creating natural convection in vertical parallel plate microchannels with uniform and asymmetric heat flux in the walls. The research is conducted for various amounts of *Ha*, M, *Gr*, *Kn*, and rq to find their influence on temperature profile, velocity profile, Nusselt number, and mass flow rate.

It is carried out for different values of *Ha*, M, *Gr*, *Kn*, and the rq to find their effects on the velocity and temperature profiles, Nusselt number, and mass flow rate. It wishes that the results be benefitcial to understand the effects of magnetic field on natural convection gas slip flow and useful to help the fabrication technology of microfluidic system devices.

## 2 Problem statement

**Fig. 1:**
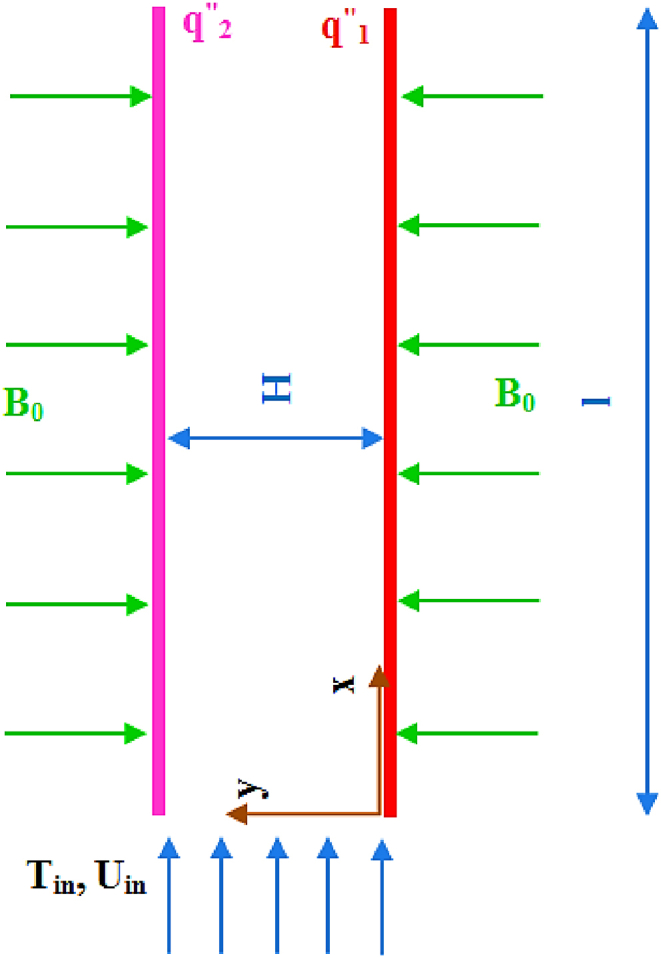
Channel geometry.

This research is aimed at investigating the MHD free convection gaseous slip flow in a vertical parallel plate microchannel supposing that Pr = 0.7. The nanochannel walls have uniform and asymmetrical heat flux. Fig. 1 depicts the channel geometry. x and y respectively indicate horizontal and vertical coordinates, which are measured from the channel inlet section. This study investigates the flow numerical simulation in the limit of 0≥ M ≥ 2; 0≥*Kn* ≥ 0.1; 0.1≥ rq ≥ 1 and 0.0001≥ L ≥ 0.01 has been investigated. The flow is subjected to a uniform lateral magnetic field, $B_{0}$. It is noteworthy that because, since the magnetic field along with the magnetic Reynolds number is insignificant, the induced magnetic field caused by the movement of conducting fluid is unimportant and could be neglected. Furthermore, Hall effects and joule heating are neglected.

## 3 Equations of motion

The flow is supposed to be incompressible, two-dimensional, laminar, and steady with temperature-independent thermo-physical properties except for density. Also, viscose dissipation is negligible. Under the above assumptions, by applying Boussinesq approximation, mass, momentum, and energy governing equations will be [37,38]:

(1) $$\frac{\partial u}{\partial x}+\frac{\partial v}{\partial y}=0$$

(2) $$\frac{\partial u}{\partial t}+\rho \left(u\frac{\partial u}{\partial x}+v\frac{\partial u}{\partial y}\right)=\frac{-\partial p}{\partial x}+\frac{\partial }{\partial x}\left(\mu \frac{\partial u}{\partial x}\right)+\frac{\partial }{\partial y}\left(\mu \frac{\partial u}{\partial y}\right)+\rho g\beta \left(T-T_{0}\right)-\sigma B_{0}^{2}u$$

(3) $$\frac{\partial v}{\partial t}+\rho \left(u\frac{\partial v}{\partial x}+v\frac{\partial v}{\partial y}\right)=\frac{-\partial p}{\partial y}+\frac{\partial }{\partial x}\left(\mu \frac{\partial v}{\partial x}\right)+\frac{\partial }{\partial y}\left(\mu \frac{\partial v}{\partial y}\right)$$

(4) $$\frac{\partial T}{\partial t}+\rho cp\left(u\frac{\partial T}{\partial x}+v\frac{\partial T}{\partial y}\right)=\frac{\partial }{\partial x}\left(k\frac{\partial T}{\partial x}\right)+\frac{\partial }{\partial y}\left(k\frac{\partial T}{\partial y}\right)$$

Where μ refers to dynamic viscosity, p indicates pressure, β is volumetric thermal expansion coefficient, $B_{0}$ implies uniform magnetic field strength, σ is the fluid electrical conductivity, cp is the specific heat, K the thermal conductivity, T the temperature and t indicates time. $-\sigma {B_{0}}^{2}u$, is often known as the Lorentz force. Applying a first-order model, the slip velocity could be defined as described below [39]:

(5) $$u_{s}=\frac{-2-\sigma_{v}}{\sigma_{v}}\;\lambda \;\frac{\partial u}{\partial y}{|}_{wall}$$

The Jump temperature at the wall could also be defined as [39]:

(6) $$T_{gas}-T_{wall}=-\frac{2-\sigma_{T}}{\sigma_{T}}.\frac{2\gamma }{\gamma +1}.\frac{\lambda }{\mathrm{pr}}\;\frac{\partial T}{\partial y}{|}_{wall}$$

Here $U_{s}$ implies the slip velocity, λ the molecular mean free path. $\sigma_{T}$ and $\sigma_{V}$ indicate the coefficients of thermal accommodation and tangential momentum, respectively. These values could be obtained empirically. In the above equation, γ is the specific heat ratio, and $T_{gas}$ is the gas temperature. $\sigma_{V}$ and $\sigma_{T}$ depend on the type of the gas as well as the material of the surface; however, in this work, these values are assumed equal to one [39]. To present results the following dimensionless variables are used [38,39].

(7) X=xGrl,Y=yH,U=H2uνGrl,V=Hvν,θ=T−T0q1Hk,Gr=gβq1H5kν2l,M=(σB02H2μ)1/2,Kn=λH,L=1GrNu1(x)=h1(x)Hk=q1H(T1,w(x)−Tin)k,Nu2(x)=h2(x)Hk=q2H(T2,w(x)−Tin)k,m=UinH2lνGr=∫01UdY=Uin,Nu‾=q‾b(Tw,ave−T0)k=2θ‾1.1/2+θ‾2.1/2,θ‾=21+rqθ,m‾=21+rqm,q‾=q1+q22q,rq=q1q2,τ=αtH2

Where, ½ points out the values at X=L/2.

## 4 The solution method and data production

### 4.1 Numerical method

The governing equations (1), (2), (3), (4) could be solved through applying boundary conditions (7–9) using a finite volume method. The SIMPLEC co-located body-fitted algorithm is used. Convection terms are discretized with the QUICK scheme, while for diffusion terms central differencing method is used. An iterative procedure with the convergence criteria of 1 × 10^−6^ for energy and 1 × 10^−6^ for velocity and momentum components (x and y axes) is applied in order to achieve a proper convergence The time to perform computational operations for the convergence of each of the examined states is around 20–30 min. The numerical code that is used to solve the governing equations, has the following steps:

1 Chose values for Kn, Pr, and Gr
2 Guess the value for $u_{in}$
3 Solve equations (1-4) imposing the boundary conditions (7–9) using the SIMPLEC algorithm to calculate velocity, temperature, and pressure profiles (u, v, T, and P)
4 Calculate pressure at the channel outlet section and call it $p_{out}$
5 Repeating steps 2–4 for the new value of $u_{in}$ and calculate the new value for $p_{out}$.
6 Consider a function between $p_{out}$ and ${\;u}_{in}$ from the value, values of steps 4 and 5. Employing the Newton- Raphson method to find the new value for $u_{in}$ and then, calculate $p_{out}$ again. This process should be repeated until the outlet channel pressure is obtained to be equal to zero.
7 Repeat steps 2–6 for the next time step, until the steady state condition is reached.

### 4.2 Boundary conditions

The systems of equations (4), (5), (6) could be solved by applying these boundary conditions [40]:

**Inlet** [40]**:**

v=0, $u=u_{in}$, $T=T_{in}$ , $p=-\frac{1}{2}u_{in}^{2}$ , Where $u_{in}$ is the uniform and constant velocity of gas that enters the channel and T_in_ is the gas temperature at the channel inlet.

**Outlet:**$P=P_{0}$, where $P_{0}$ is the ambient pressure at the channel outlet section.

**At right wall** [40]**:**

v=0

(8) u=2−σvσvKn.H∂u∂y|y=0,∂T∂y|y=0=−q1k″

**At left wall** [40]**:**

v=0

(9) u=−2−σvσvKn.H∂u∂y|y=H,∂T∂y|y=H=−q2k″

### 4.3 Solution assumptions

The flow is subjected to a uniform lateral magnetic field $B_{0}$. It is noteworthy that due to the fact that the magnetic field along with the magnetic Reynolds number is insignificant, the induced magnetic field caused by the movement of conducting fluid is unimportant and could be neglected.

### 4.4 Grid independency

In order to verify spatial grid independence, the numerical code was carried out for a variety of nodes in x and y directions for *Kn* = 0 and L = 0.0001. The quantity is chosen to examine grid independency are average Nusselt number. The results are shown in Table 1. It is revealed that there is very small error between the results at grids holding 100 × 6o nodes and 150× 70 nodes. Consequently, a grid system with 100 rows and 60 columns is used for computational purposes.

**Table 1:** Grid study results.

Grid resolution	*Nu*
20×20	3.989
30×30	4.098
100×60	4.238
150×70	4.239
Ref [ 38]	4.1

**Fig. 2:**
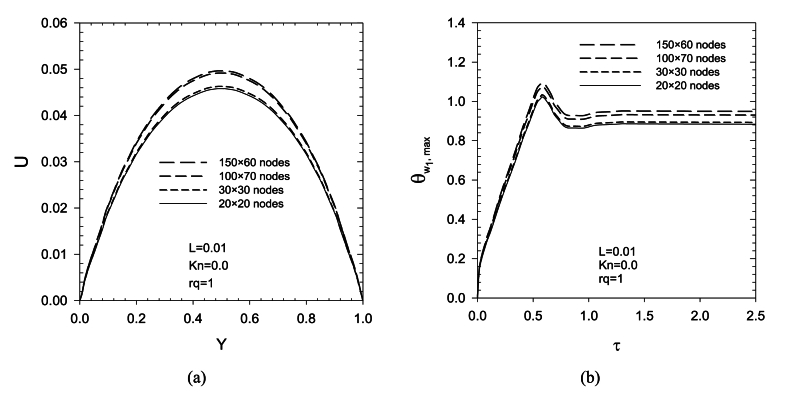
Examining the grid study in the present research with the examination of (a). dimensionless velocity profile and (b). variations of the maximum dimensionless temperature of the wall 1.

The grid study in Fig. 2(a, b) has also been investigated for dimensionless velocity changes and maximum dimensionless temperature changes on wall 1. Considering the changes in the number of grides and its effect on the investigated parameters, by choosing the number of grades 100 × 70, one can be sure of the validity of the results. Therefore, the simulation of the present research is done with this number of grides.

## 5 Results and discussions

### 5.1 Code validation

**Fig. 3:**
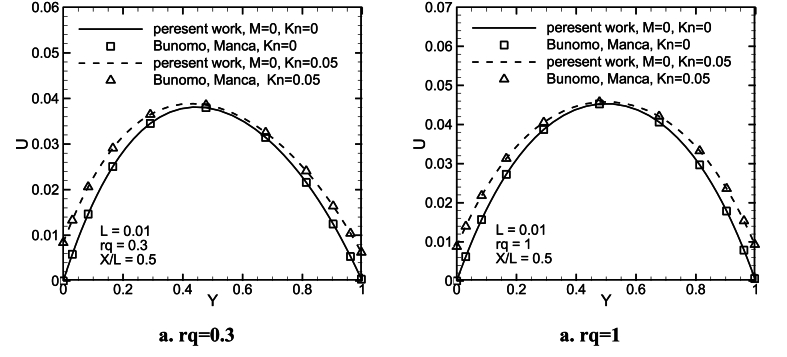
A comparison of non-dimension velocity profiles with the values of Buonomo and Manca [38], for L = 0.01, *Kn* = 0, 0.05, and rq = 0.3, 1, at X/L = 1/2.

**Fig. 4:**
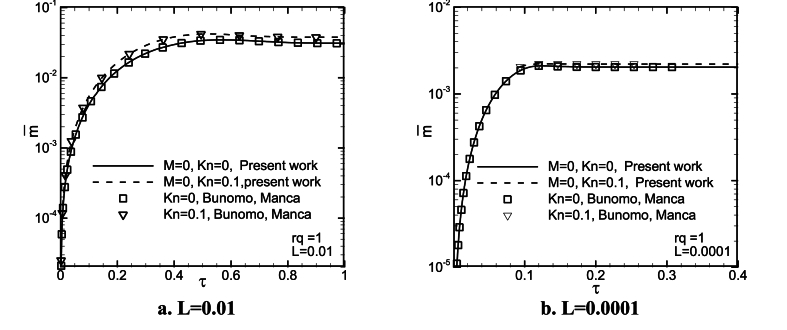
A comparison of the non-dimension mass flow rate as a function of time with those of Buonomo and Manca [38], for L = 0.01, 0.0001, *Kn* = 0, 0.1, and rq = 1.

To verify the numeric code, the steady-state velocity profiles, average Nusselt number, the dimensionless mass flow rate as a function of time are compared with those numerical solutions given by Buonomo and Manca [38], for L = 0.01, 0.0001, *Kn* = 0, 0.05, 0.1, and rq = 0.3, 1. The corresponding results are shown in Fig. 3(a, b) and Fig. 4(a and b), and tabulated in Table 2, Table 3. As can be observed, they conform well with the values from Buonomo and Manca, except for some cases for average Nusselt numbers.

**Table 2:** A comparison of the average Nusselt number from the current study with Ref [38], rq = 1.

Kn	Gr	Present work $\bar{Nu}$	Ref [38] $\bar{Nu}$
0	100	1.72	1.79
0.1	100	1.34	1.79
0	10000	4.24	4.1
0.1	10000	2.81	2.79

**Table 3:** Comparison between average Nusselt number from the present study with Ref [38], rq = 0. 3

Kn	Gr	Present work $\bar{\text{Nu}}$	Ref [38] $\bar{\text{Nu}}$
0	100	1.49	1.52
0.05	100	1.45	1.52
0	10000	4.0	4.0
0.05	10000	3.31	3.26

### 5.2 Mass flow rate as a function of time

**Fig. 5:**
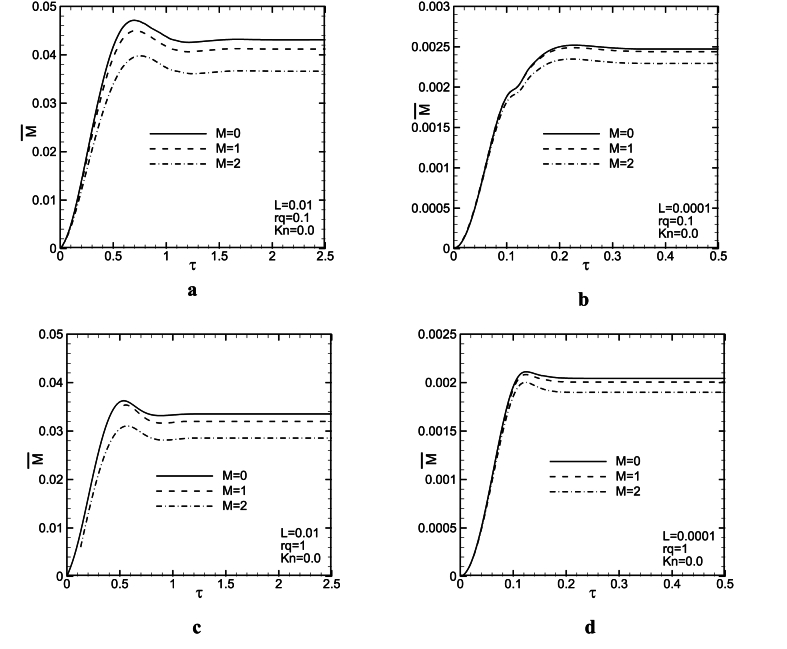
Mass flow rate as a function of time for L = 0.01, 0.0001, *Kn* = 0, and rq = 0.1, 1.

**Fig. 6:**
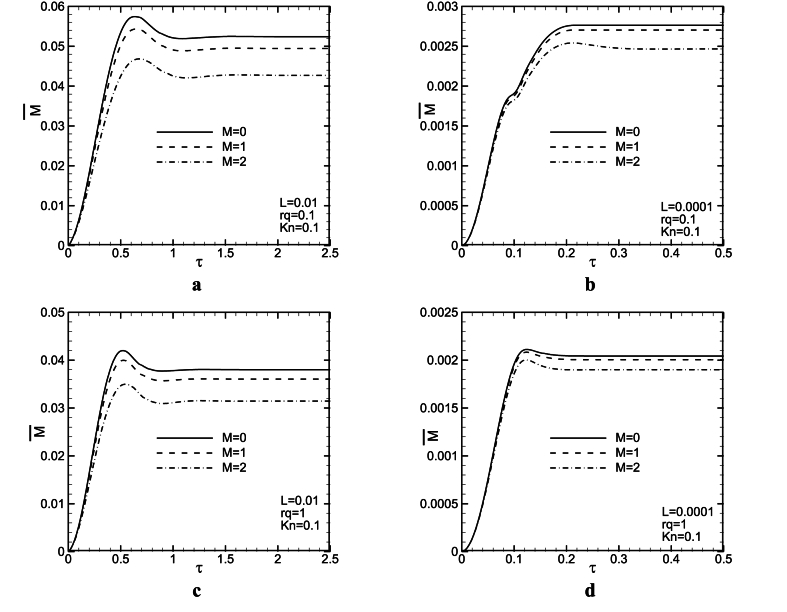
Mass flow rate as a function of time for L = 0.01, 0.0001, *Kn* = 0.1, and rq = 0.1, 1.

**Fig. 7:**
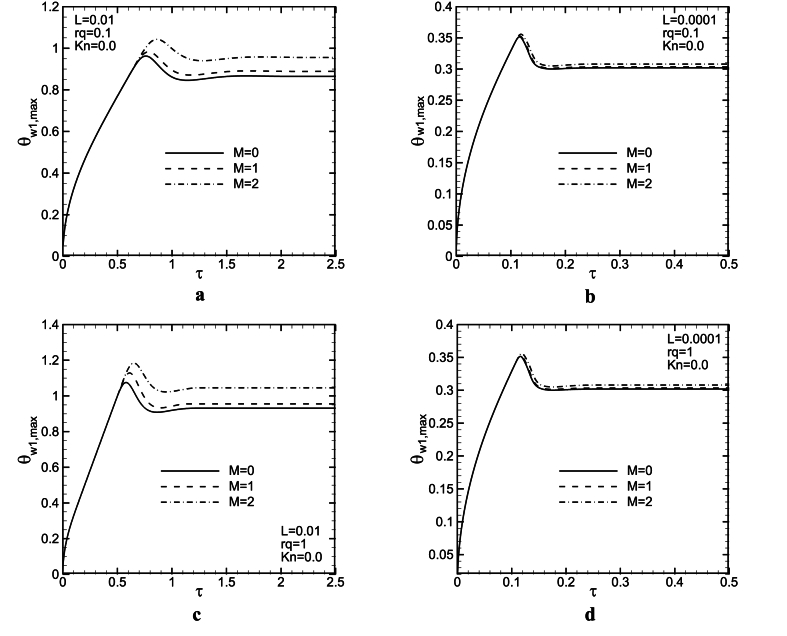
Maximum temperature of the wall as a function of time for L = 0.01, 0.0001, *Kn* = 0, and rq = 0.1, 1.

Fig. 5(a - d) and Fig. 6(a - d) are provided to provide an analysis of the influence of the magnetic field on the dimensionless mass flow rate represented as a function of time. These figures are plotted for M = 0, 1, 2 and L = 0.01,0.0001 with two rq, and *Kn* values. It could be observed from the figures that increasing the M value, decreases the mass flow rate for all rq and L values. The magnetic force is in the opposite direction of fluid motion and hence, causes a decrease in the fluid speed. Therefore, velocity and mass flow rate decrease. From Fig. 5(a–d), it is revealed that there is an overshoot in mass flow rate to the time. The overshoot decreases slightly with an increase in *Ha*. It seems, magnetic force strengthens diffusive effects and leads to weaken convective ones. Therefore, increasing M, makes diffusion terms stronger than convection ones and hence, the overshoot in mass flow rate decreases. Assuming the same conditions for parameters M and *Kn* at L = 0.01, increasing rq decreases the dimensionless mass flow rate between 16 and 21 %, and the mass flow rate decrease for the above conditions for L = 0.0001 is about 9–12 % (see Fig. 7).

Fig. 6, is plotted in the same condition as Fig. 1, but for *Kn* = 0.1. For this figure, there is a similar trend with Fig. 5(a - d). It is seen, that with increasing the Knudson number, the mass flow rate increases. This result is considered reasonable because the slip velocity in the vicinity of channel walls is increased. As the *Kn* value increases, the slip velocity will increase as well, which in turn leads to an increase in mass flow rate. In addition, at higher consider *Kn* values, the magnetic field influence on the time variations of mass flow rate is more significant. When *Kn* = 0.1, even in larger L values, magnetic force has more effect on the mass flow rate compared with *Kn* = 0. Comparing Fig. 5(a - d) and Fig. 6(a - d) revealed that in larger *Kn* values, magnetic force can change fluid behaviors better. It is noteworthy that the time in which the steady-state condition is obtained, is independent of Lorentz force. As it is remarked in Fig. 5(a - d) and Fig. 6(a - d), increasing L, leads to a significant decrease in the time at which the steady state condition is attained. In other words, as buoyancy force increases, probably, a balance between the convective and the diffusive terms is achieved sooner.

Probably, the magnetic field helps that balance between the diffusion and the convection terms is attained sooner and it is expected, in greater M values, the overshoot in mass flow rate vanishes. Also, with increasing M, the corresponding time with the overshoot, increases a little. Comparison between Fig. 5(a, c) and Fig. 5(b, d) shows that in lower L (higher *Gr*) values, the magnetic force effect lessens on mass flow rate. With an increase in *Gr*, buoyancy force increases, and hence, the convection terms become stronger than diffusion ones. Forasmuch as magnetic force probably improves the diffusion effect, and it makes convective one weak, it seems that the magnetic field influence lessens the mass flow rate by increasing *Gr*. It could be observed from Fig. 5(a, b) that at rq = 0.1, the effect of the magnetic field on the mass flow rate changes to time is a bit more.

### 5.3 Maximum temperature of the wall as a function of time

**Fig. 8:**
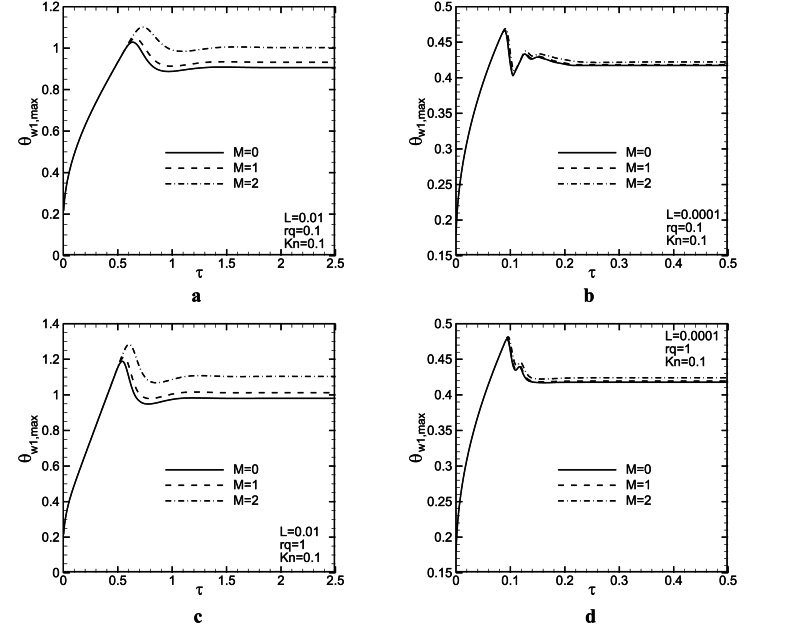
Maximum temperature of the wall as a function of time for L = 0.01, 0.0001, *Kn* = 0.1, and rq = 0.1, 1.

**Fig. 9:**
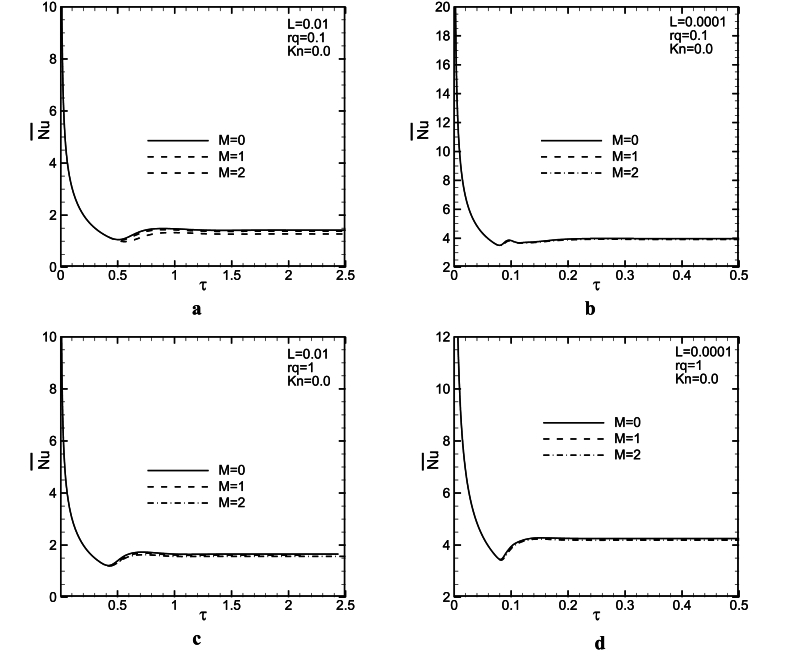
Average Nusselt number as a function of time for L = 0.01, 0.0001, *Kn* = 0, and rq = 0.1, 1.

Fig. 7(a - d) and Fig. 8(a - d) show the influence of magnetic force on the dimensionless maximum wall temperature as a function of dimensionless time at the hot wall of the channel for three M and two L values with rq = 0.1,1 when *Kn* = 0 and 0.1. It is clear that as the magnetic force increases, the maximum wall temperature increases for both L values. Increasing M, leads to a decrease in mass flow rate, and maybe, diffusion effects become stronger so that the maximum temperature of the wall increases. There is also at least one overshoot in the maximum wall temperature variations to time. In general, any factor leading to a decrease in flow axial velocity components, will cause an increase in the boundary layer velocity, hinder the movement of the flow in the microchannel, and finally result in decreased heat transfer and increased wall maximum temperature (see Fig. 9).

Fig. 8 (b, d) presents two overshoots for L = 0.0001 and *Kn* = 0.1. These overshoots decrease with an increase in M, and probably, they will vanish in greater M values. Also, the time at which the overshoot occurs, increases slightly with M. It seems, at this time, convection terms weaker than diffusion ones, but, after that, the balance between them changes. The maximum wall temperature increases with an increase in rq values. In addition, it can be seen, that when *Gr* increases (L values decrease), the maximum temperature of the wall as a function of time significantly decreases. In this case, buoyancy force increases in the channel, therefore fluid speed and maybe convection effects are strengthened so that the maximum wall temperature lessens. For lower considered L values, the magnetic field influence decreases on the maximum temperature of wall variations to time. It could be observed from Fig. 5(a - d) that as *Kn* increases, due to increases in temperature jump and slip velocity, the maximum wall temperature increases at channel walls. Fig. 8(b–d), shows an undershoot occurs after an overshoot. However, a higher *Kn* numbers, Lorentz force influence is more significant on the variations of the maximum temperature of the wall to time.

### 5.4 Average Nusselt number as a function of time

**Fig. 10:**
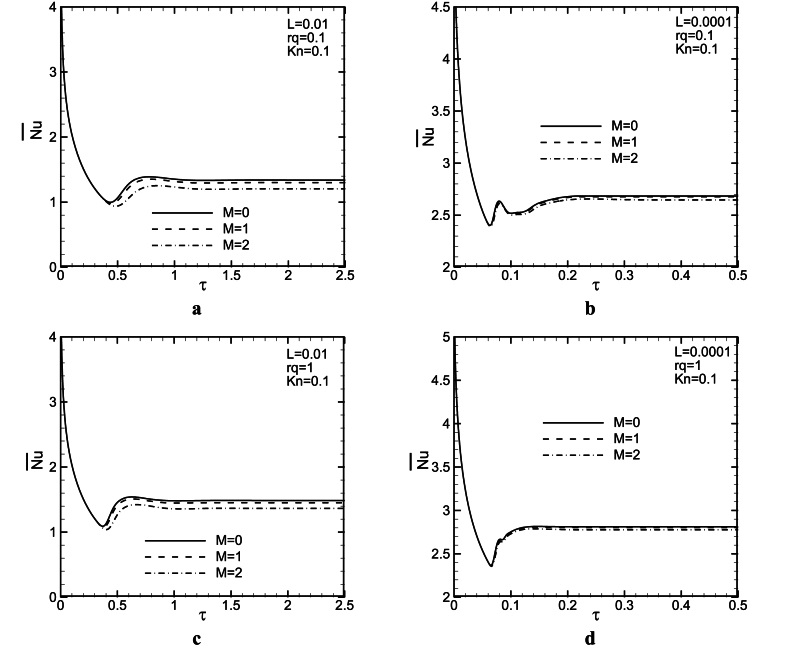
Average Nusselt number as a function of time for L = 0.01, 0.0001, *Kn* = 0.1, and rq = 0.1, 1.

Fig. 9(a - d) and Fig. 10(a - d) show the influence of the magnetic field on average Nusselt number as a function of time for M = 0, 1, 2 and L = 0.01, 0.0001, when rq = 0.1, 1 with both *Kn* = 0 and 0.1. It could be discovered that as M increases, the average Nusselt number decreases as well. In fact, increasing *Ha*, leads to an increase in the temperature of a wall along the channel, and hence, the average Nusselt number decreases.

It is also seen, that there is one or two undershoot in the average Nusselt number shown as a function of time. It is noteworthy that the time at which this undershoot presents in the Nusselt number almost corresponds with the time at which overshoot occurs in maximum wall temperature and it has a delay with the time at which overshoot is detected in mass flow rate. Also, it is revealed that when maximum wall temperature presents an overshoot, the Nusselt number shows an undershoot and vice versa. For lower L values, due to an increase in buoyancy forces, the average *Nu* increases significantly. As explained before in sections 4-1 and 4–2, buoyancy force effects, result in a significant reduction in the temperature of the wall, and hence, as *Gr*. increases, the average Nusselt number rises as well (lower L values). In contrast to the mass flow rate and maximum temperature of the wall that are significantly affected by magnetic force, the magnetic field has less influence on the average *Nu* number.

### 5.5 Velocity profile

**Fig. 11:**
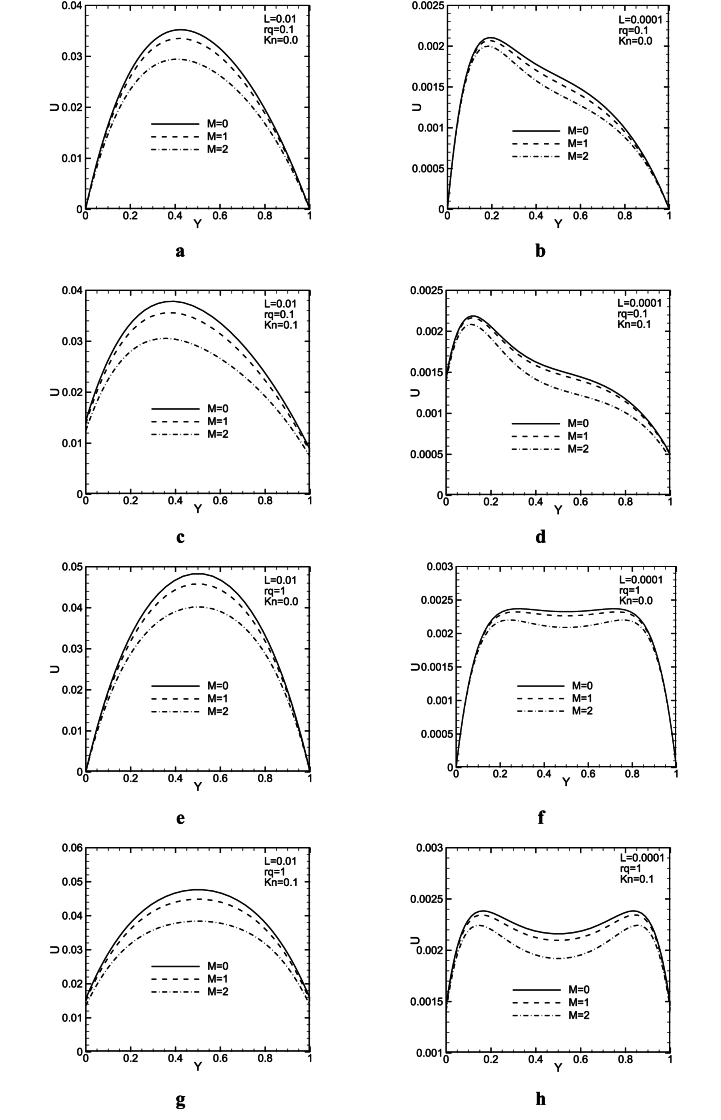
Dimensionless velocity profiles for different M, *Kn*, and rq at X/L = 1/2.

Fig. 11(a - h) shows different amounts of *Kn*, *Kn*, *Ha*, M, rq, and dimensionless channel height, L for analyzing the influence of the magnetic field on the dimensionless profile of velocity. It is clear that as M increases, the velocity diminishes f for both L = 0.01 and L = 0.0001 values. The magnetic force is in the opposite direction of fluid motion which consequently reduces the channel velocity. For lower L values, buoyancy force increases in the channel and hence, the convective effect becomes stronger than the diffusive one. Therefore, the magnetic force effect lessens the velocity. As Fig. 11(c, d) show as L increases, the velocity in the vicinity of the hot wall increases as well. Furthermore, increasing the *Kn* value results in increased slip condition which in turn makes buoyancy force effects more important in the vicinity of channel walls.

Also, an increase in *Kn* leads to an increase in slip condition and hence, becomes more significant near the channel walls. Moreover, a comparison between the velocity for different *Kn* shows that at higher *Kn* values the effect of parameter M, is more significant for both L = 0.01 and 0.0001.

### 5.6 Temperature distribution

**Fig. 12:**
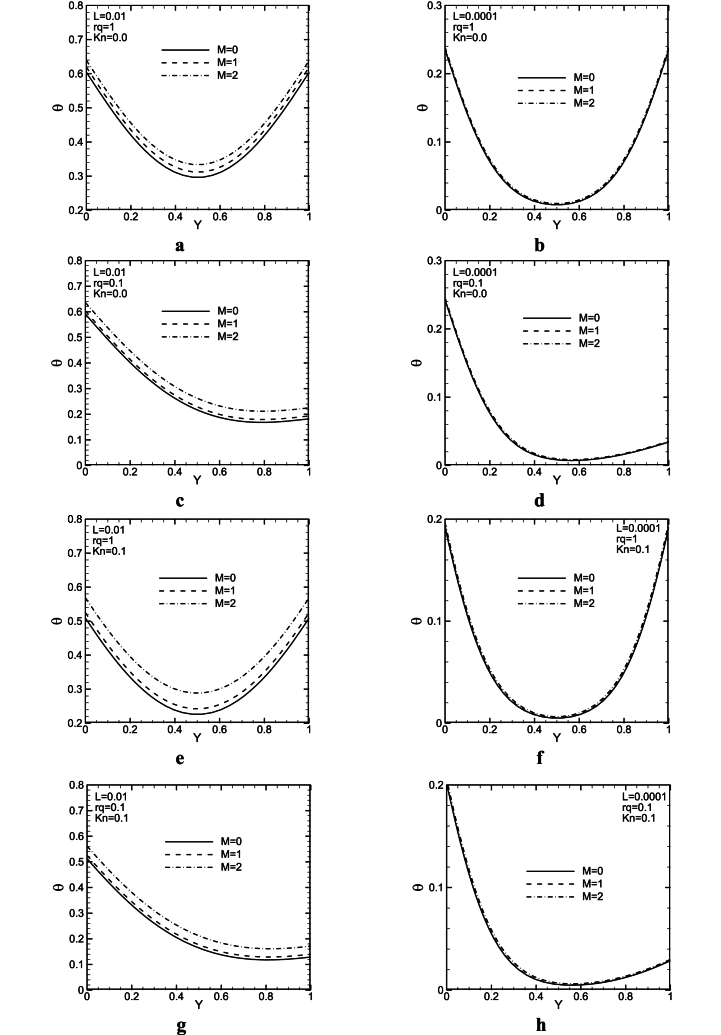
Dimensionless cross-section temperature profiles for different M, *Kn*, and rq at X/L = ½

Fig. 12(a - h) shows the effect of the magnetic field on the dimensionless temperature, for rq = 0.1 and 1, with *Kn* = 0, 0.1, and three values of *Ha* at $\frac{X}{L}=0.5$. It is revealed from Fig. 12(a–h) that cross-section temperature is affected more significantly by magnetic force for L = 0.01 to the L = 0.0001. In greater *Gr* values (smaller L), buoyancy force increases and it makes the convective effect stronger than the diffusive one. Forasmuch as magnetic force probably improves diffusion effect, and it makes convective one weak, it seems, the magnetic field influence lessens on profiles of temperature with an increase in *Gr*. For L = 0.0001 in Fig. 12(b–d, f, h), it is observed that the magnetic field is of great influence on the profile of velocity; however, it has very little influence on temperature profiles. For higher *Kn*, due to an increase in temperature jump, the Lorentz force effect increases on the temperature.

## 6 Conclusion

This research presents a numerical study over unsteady laminar flow and natural convection of an electrically conducting fluid in a vertical parallel plate microchannel under uniform and asymmetric heat flux subjected to a uniform lateral magnetic field. Slip velocity, as well as temperature jump at channel walls, are modeled using a first-order model. To solve equations of motion, The SIMPLE-C Co-Located scheme with a Newton-Raphson method is used. Effects of *Ha*, M, *Gr*, rarefaction of gas, *Kn*, and heat flux

on the mass flow rate, maximum temperature wall, and average Nusselt number changes to time were studied. In this research, the flow in the limit of 0≥ M ≥ 2; 0≥*Kn* ≥ 0.1; 0.1≥ rq ≥ 1, and 0.0001≥ L ≥ 0.01 were simulated numerically. Variations of profiles of steady-state velocity and temperature are also discussed. It was concluded that the.

Mass flow rate as a function of time presents an overshoot. With the increase of *Ha*, the overshoot reduces. Moreover, increasing M results in a reduction in velocity and mass flow rate. Maximum wall temperature increases with an increase in M. Assuming the same conditions for parameters M and *Kn* at L = 0.01, increasing rq decreases the dimensionless mass flow rate between 16 and 21 %, and the mass flow rate decrease for the above conditions for L = 0.0001 is about 9–12 %.

There is an overshoot in the maximum temperature of the wall to time. As M rises, the overshoot decreases. In general, any factor causing a reduction in flow axial velocity components, increases the boundary layer velocity, hinders the movement of the flow in the microchannel, and will ultimately reduce the heat transfer and increase the maximum temperature of the wall. The average Nusselt number decreases with increasing M. In contrast to temperature, The *Nu* number detects an undershoot in time. The magnetic force influence on profiles of velocity and temperature profiles decreases as *Gr*. increases. For higher *Kn* values, the magnetic force effect increases on the fluid and thermal behaviors.

Then, the effect of turbulence in this geometry can be studied for high Rayleigh numbers or the presence of roughness or sectional narrowness in the microchannel using Newtonian and non-Newtonian nanofluids.

**Table:** 

**Nomenclature**			
$B_{0}$	magnetic field strength, Tesla	**Greek symbols**	
$c_{p}$	specific heat at constant pressure, J/kgk	α	thermal diffusivity, m2s
g	gravity, $m/s^{2}$	γ	specific heat ratio
Gr	Grashof number, Eq. (10)	λ	molecular mean free path, m
l	channel height, m	μ	dynamic viscosity, pa.s
L	dimensionless channel height, Eq. (10)	ρ	density, $Kg/m^{3}$
H	distance between plates, m	σ	electrical conductivity, 1/Ωm
K	thermal conductivity, $W/m^{2}k$	$\sigma_{t}$	thermal accommodation coefficient
Kn	Knudsen number, λ/H	$\sigma_{v}$	tangential momentum accommodation coefficient
M	Hartmann number, Eq. (10)	ν	kinematic viscosity, $m^{2}/s$
m	dimension less mass flow rate	θ	dimensionless temperature, Eq. (10)
Nu	Nusselt number, Eq. (10)	η	magnetic diffusivity
p	pressure, pa	τ	dimensionless temperature, Eq. (10)
pr	Prandtl number, ν/α		
$r_{q}$	heat flux ratio, Eq. (10)	**Subscripts**	
q	heat flux, W/mk	a	average
$q_{1}$	heat flux at the right channel wall	s	slip/jump values
$q_{2}$	heat flux at the left channel wall	w	wall values
Re	Reynolds number	½0.1	midpoint of left wall channel
${Re}_{m}$	magnetic Reynolds number, uH/η	½0.2	midpoint of right wall channel
T	temperature, K	in	inlet section of channel
$T_{w}$	wall temperature	−	a bar is used for the quantities in Eq. (10)
u,v	velocity components in the x and y direction, ms		
U,V	dimensionless velocities, Eq. (10)		
x,y	axial and normal coordinates, m		
X,Y	dimensionless coordinates, Eq. (10)		

## CRediT authorship contribution statement

**Mohsen Saghafian:** Writing – review & editing, Writing – original draft, Visualization, Validation, Supervision, Resources, Methodology, Investigation, Funding acquisition, Formal analysis, Data curation, Conceptualization. **Mehdi Moslehi:** Writing – original draft, Validation, Resources, Methodology, Funding acquisition, Data curation. **Omid Ali Akbari:** Writing – review & editing, Writing – original draft, Visualization, Validation, Supervision, Software, Resources, Project administration, Methodology, Investigation, Funding acquisition, Formal analysis, Data curation, Conceptualization.

## Declaration of Competing Interest

The authors declare that they have no known competing financial interests or personal relationships that could have appeared to influence the work reported in this paper.
